# Sugar-sweetened beverages consumption among New Zealand children aged 8-12 years: a cross sectional study of sources and associates/correlates of consumption

**DOI:** 10.1186/s12889-021-12345-9

**Published:** 2021-12-13

**Authors:** Emma Smirk, Hajar Mazahery, Cathryn A. Conlon, Kathryn L. Beck, Cheryl Gammon, Owen Mugridge, Pamela R. von Hurst

**Affiliations:** 1 School of Sport, Exercise and Nutrition, College of Health, Massey University, Auckland, 0745 New Zealand; 2 School of Health Sciences, College of Health, Massey University, Auckland, 0745 New Zealand

**Keywords:** Sugar sweetened, Beverage, Consumption, Knowledge, Children

## Abstract

**Background:**

The benefit of reducing sugar-sweetened beverage (SSB) consumption is widely accepted, but updated and in-depth data on New Zealand (NZ) children’s SSB consumption is lacking. The aims of this study were to describe beverage consumption, focusing on SSBs in primary school age children living in Auckland; to examine the association of selected socio-demographic, home, community and school factors and children’s beverage knowledge/attitudes with regards to beverage consumption; to explore the relationship between SSBs consumption and adiposity in children.

**Methods:**

A cross-sectional, Auckland-wide survey of 578 school age children (8-12 years) was conducted using questionnaires to collect data on beverage consumption, beverage knowledge/attitudes, and selected socio-demographic and home, community, school factors. Body fat percentage (BF%) was assessed using bioelectrical impedance (BIA).

**Results:**

Ninety-six percent of children consumed ≥1 serving of SSBs a week; with ≥5 servings reported by 62% of children. Of all SSBs assessed, consumption of ≥1 serving of sugar sweetened milk-based beverages (85%, mainly milk drinks made from powder) was most prevalent, followed by fruit juice (46%) and sugar-containing carbonated drinks (39%, mainly soft/fizzy drinks). Among unsweetened beverages, plain water was reported to be consumed < 2 times a day by 22% of children, and plain milk < 1 serving a day by 53%. Higher consumption of SSBs was associated with socio-economic disadvantage, non-NZ European ethnicities (Māori, Pacific, Asian, others), availability of SSBs in the home, frequent takeaway/convenience shop visits, children’s incorrect perception of adequate SSBs consumption frequency, and higher BF% (females only). School health policy and encouragement of children to consume un-sweetened beverages was not associated with SSBs consumption.

**Conclusions:**

The consumption of SSBs is prevalent in NZ school age children, with higher consumption rates observed among those from socially disadvantaged areas. This high consumption is associated with higher BF% in females. Multi-contextual interventions to decrease SSBs should target children, and their families/environment, particularly those from socially disadvantaged areas.

**Supplementary Information:**

The online version contains supplementary material available at 10.1186/s12889-021-12345-9.

## Background

Despite the issue of childhood obesity being at the forefront of policy makers’ minds, a large proportion of New Zealand (NZ) children are overweight (20%) and obese (12%) [1–3]. This rate has not changed significantly since 2011/2012, and is disproportionately higher in some ethnic groups (e.g. 29 and 13% in Māori and 31 and 29% in Pacific children, respectively) and low socio-economic areas (almost three times as likely to be obese as children living in the higher socio-economic areas) [3]. This high prevalence of overweight/obesity is a major public health concern because of associated physical comorbidities and psychological complications partly due to discrimination against people of higher weight [4–6]. Obesity is multifactorial in origin, with sugar-sweetened beverages (SSBs) consumption being recognised as an important risk factor [7, 8]. Sugar-sweetened beverages are any beverages that contain added caloric sweetener, usually sugar (e.g. sucrose, glucose, lactose, maltose, and fructose). Sugars in a liquid form may induce less satiety than in a solid form [9] and may promote the over-consumption of energy, contributing to the development of obesity and excess adiposity, and associated comorbidities (e.g. metabolic syndrome and diabetes).

The link between excessive SSBs consumption and childhood obesity has been repeatedly, though inconsistently, reported in the literature [10, 11]. A review of meta-analyses by Keller et al. [10] showed that while some studies demonstrated a positive association between SSBs consumption and weight gain, overweight, or obesity in children, others failed to replicate such findings. A systematic review by Frantsve-Hawley et al. [12] showed the consumption of SSBs was positively associated with total adiposity among children < 5 years old, but for children < 12 years old the evidence was mixed. Regardless, most studies suggest an overall increased risk of obesity and higher adiposity with SSBs consumption. This has led international (World Health Organisation, WHO) and national health authorities (e.g. New Zealand Ministry of Health, NZ MOH) to include the reduction of SSBs consumption in obesity prevention strategies [2, 13].

The NZ MOH recommends that plain water and plain low-fat milk should be consumed daily and make up most of a child’s fluid intake. They also recommend that if SSBs are consumed they are limited to less than once a week [13]. Based on the WHO recommendation, added sugars should contribute less than 10% of total energy [14]. The 2002 New Zealand National Children’s Nutrition Survey found SSBs to be one of the largest contributors of added sugar to the diets of New Zealand children [15], with disproportionally higher consumption rates reported in boys (33% as opposed to 24% in girls), and some ethnic groups (e.g. 49% in Pacific and 39% in Māori children) [16]. As far as we are aware the latest data on a range of beverages, but not SSBs, in the 8 to 12 year old age group (*n* = 454) was published more than 10 years ago in the Children’s Food and Drinks survey, and showed that 51 and 35% of children consumed ≥1 serving of fruit juice and sugar-containing carbonated drinks a week, respectively [17].

Evidence suggests that early childhood is a critical period of life [18] where unhealthy dietary habits such as SSBs consumption can be formed. Therefore, it is of the utmost importance to understand factors influencing children’s SSBs consumption to inform effective intervention programs. Several studies have been conducted with parents and teachers of children, adolescents, and young adults to elucidate factors influencing SSBs consumption and to investigate knowledge and attitudes toward SSBs consumption [19–25]. However, to date, to the best of our knowledge, no study has assessed this in New Zealand children.

Therefore, the aims of this study were to describe beverage consumption, focusing on SSBs, in primary school age children living in Auckland; to examine the association of selected socio-demographic, home, community and school factors and children’s knowledge/attitudes with regard to beverage consumption; and to explore the relationship between SSBs consumption and adiposity in children.

## Methods

### Study design and participants

Data for this study were drawn from an Auckland-wide observational, cross-sectional study named ‘Children’s Bone Study’. Accordingly, no sample size calculation was performed for this study. School children aged 8 to ≤12 years from throughout Auckland were enrolled (August to September 2016 and 2017, winter season).

Six primary schools participated in the study, of which four were water-only schools (schools which only allow the sale of water and plain milk on school property) and two were not. School deciles were used as a proxy of the children’s socio-economic status, with a low decile representing a low socio-economic status. Schools were specifically included to provide a range of socio-demographic levels and ethnicities. Schools were approached through a collaboration with primary school science teachers and asked for expressions of interest. All children within the age group specified, and attending the school were invited to participate, and those with a history of any disease affecting calcium and vitamin D metabolism, gastrointestinal disorders, long-term medication (e.g. corticosteroids, anticonvulsants and immune-suppressants) use, or any surgical implants, metal screws or similar were excluded.

### Data collection

#### Questionnaires

Data was collected via paper questionnaires, including demographics (age, sex, ethnicity, and name of participating school), beverage type and frequency, and beverage knowledge and attitude questionnaires, all of which were completed at home. Whilst the demographics questionnaire was directed to parents/caregivers, the beverage type and frequency questionnaire was asked to be completed by children with help from their parents/caregivers. The beverage knowledge and attitude questionnaire was to be completed by the children themselves. All questionnaires were designed by researchers for this study and have not been validated.

The beverage type and frequency questionnaire asked about the type and frequency of different beverages consumed and was modelled on the food frequency questionnaire used in New Zealand National Nutrition Survey 2002 [15] (see Table 1 and Supplementary material 1). Selected questions about home, school and community factors were also included in this questionnaire. The total unsweetened beverages, all SSBs, and sugar containing carbonated beverages were calculated as below:

- Total SSBs: All beverages containing added sugars (e.g. fruit drinks, flavoured milk, soft/fizzy drinks). Because children cannot distinguish fruit juice from 100% fruit juice and whether fruit smoothies contain added sugar or not, these beverages were not included in SSBs category and are reported separately.
- Total sugar containing carbonated beverages: Soft drinks + soda stream drinks + energy drinks

**Table 1:** Type of beverages and frequency patterns included in the “Beverage Type and Frequency Questionnaire”

Beverages	Consumption frequency A serving size: one glass
Flavoured milk	∙ Never or less than once a month ∙ 1-3 times a month ∙ 1-2 times a week ∙ 3-4 times a week ∙ 5-6 times a week ∙ Once a day ∙ 2 or more times a day
Milkshake or milk drink	
Flavoured powdered milk drink	
Powdered fruit drink	
Fruit drink concentrate/cordial	
Flavoured water	
Soft/fizzy drink	
Soda stream drinks	
Energy drinks	
Sports drinks	
Tea/coffee (sweetened)	
Fruit juice	
Fruit smoothie	

To create the grouped beverage variables, nominal consumption variables were scaled, and then added together. This potentially underestimated beverage consumption, as the lower value of each beverage frequency was used. For example, one to two times per week was scaled to once per week. Total and individual sweetened and sugar containing carbonated beverages were categorised as < 1, 1-4, and ≥ 5 servings a week. The reason for this categorisation method was that the consumption of SSBs are recommended to be limited to less than 1 per week [13].

The beverage knowledge/attitudes questionnaire included six major questions which asked children to:

- Identify how often each drink should be consumed by using a likert-scale question (drink every day or most days, sometime, special occasions, not at all, don’t know). Sugar-sweetened beverage categories included in this question were fizzy drinks, sweetened drinks, sports drink/ water or flavoured water, energy drinks, flavoured milk. Fruit juice and smoothie were also included in this question, but because children cannot distinguish fruit juice from 100% fruit juice and whether sugar is added to fruit smoothie or not, the data are not reported here.
- Rate the healthiness of certain drinks, by putting drinks in the order of their healthiness, with 1 being the most healthy and 5 the least healthy
- Identify the type of sugar in drinks by putting a tick in the box that the child thinks fits best (sugar sweetened/has sugar added, naturally sweet, non-sweetened drink/no sugar added, don’t know)
- Indicate the reasons for choosing a drink while out (multiple answers were permitted; satisfying thirst, being healthier than snacks, better for me, home/school permission, friend influence, affordability, taste)
- State how much children and their friends care about drinking healthy beverages (not at all, sometimes, a lot)
- State how much school and home were encouraging children to drink healthy beverages (not at all, sometimes, a lot)

Children were encouraged to answer all questions and were asked to give the answer that best explains what they think, not what others think. When asking about a particular beverage category knowledge/attitude, photos of beverages within that category (as examples) were provided. Also, a “don’t know” option was included to avoid random answers.

#### Anthropometrics and body composition

Anthropometric (waist circumference, weight and height) and body composition measurements were obtained during the school visits by trained personnel. Waist circumference was measured in duplicate using the landmarks for waist measurements with a Lufkin W606PM pocket tape positioned around the body over light clothing whilst standing and recorded to the nearest 0.1 cm. The mean height was calculated without shoes from two measurements using a portable stadiometer (Seca 213). Bioelectrical impedance analysis (BIA) (Biospace InBody 230) was used, without shoes and in light clothing, to measure the children’s weight and body fat percentage (BF%). BF% measurements using the BIA have been validated against the DEXA for NZ children aged 8 to 13 [26]. Body fat (BF)% was then categorised into two groups based on the median BF% of the present study population; < 21% and ≥ 21%. Body mass index (BMI) was calculated from the measurements of weight and height; weight/height,^2^ kilogram (kg)/metre (m).^2^

### Statistical analysis

Statistical analyses were performed using IBM SPSS version 27.0 (IBM Corp; Armonk, NY, USA). A *P*-value of < 0.05 was considered significant. Descriptive statistics were used to report the proportion of children consuming SSBs and sugar containing carbonated beverages, as well as each beverage type individually. The relationship between categorical variables (sex, ethnicity and school decile, BF% categories, beverage knowledge/attitude variables) and beverage consumption was investigated using Chi-square analysis. Continuous variables (e.g. height, weight, waist circumference, BMI, and BF%) were reported as median (25th, 75th percentiles) and compared across beverage consumption categories using Kruskal-Wallis test (as testing showed the data was non-normally distributed). Age was reported as mean ± SD and was compared across beverage consumption categories using one-way analysis of variance (ANOVA).

Association of SSBs consumption with BF% was assessed using binary logistic regression analysis (univariable and multivariable). To avoid the violation of multicollinearity and incomplete information from the predictors (due to many variables with many categories) and because there was a strong relationship between ethnicity and school decile (a smaller proportion of children of European ethnicity were from low and medium decile schools; 26% vs. 73-84%, *P* < 0.0001), we ran two regression analyses including either ethnicity or school decile to investigate the relationship. As the inclusion of either of these variables did not affect the results, the results with school decile are reported. The models were investigated to determine if all the assumptions were met and which model had a better model fit (assessing − 2 log likelihood). We also added interaction terms into the models to investigate for interaction effects between variables (included in the model), but no significant results were observed. Associations were described using adjusted odds ratios (OR) and 95% confidence intervals (CI).

## Results

### Children’s characteristics

**Fig. 1:**
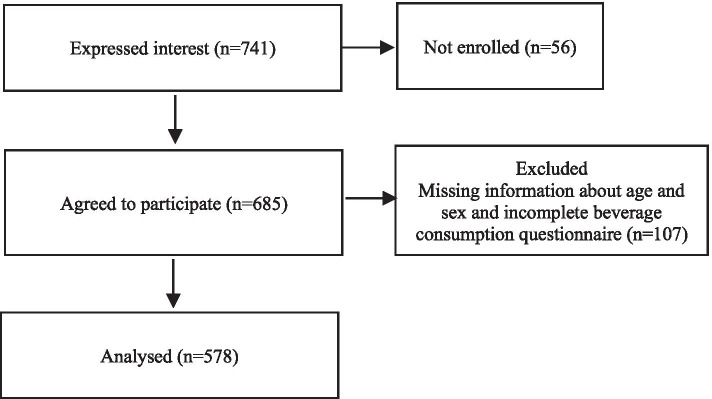
Study flow diagram

Of the 741 children invited to participate in the wider Children’s Bone Study, 685 agreed to participate in the study. Of 685 children, 107 were excluded due to missing information about age and sex and incomplete beverage type and frequency questionnaire (≥1 missing answer), leaving 578 children for the final analysis (Fig. 1). The socio-demographic characteristics of included versus excluded children did not differ significantly.

Children’s characteristics are presented in Table 2. The proportion of children from low, medium and high decile schools was 26, 33 and 41%, respectively. The mean ± SD age of children was 9.9 ± 0.7 years, and a slightly larger proportion of children were girls (54%). A range of ethnicities were present, including European (41%), Māori (10%), Pacifica (21%), Asian (24%), and other ethnicities (4%). The median (25th, 75th percentiles) BF% of children was 21 (16, 28) %.

**Table 2:** Participants’ characteristics by sugar-sweetened beverages consumption categories

	Total ***n*** = 578	Sugar-sweetened beverages (Servings per week)			***P***-value ^**a**^
		< 1 serving ***n*** = 23	1-4 servings ***n*** = 266	***≥5 servings n = 289***	
Age (y), mean ± SD	9.9 ± 0.7	9.8 ± 0.7	9.8 ± 0.7	9.9 ± 0.7	0.32
Sex, n (%)					0.16
Boys	265 (46)	8 (3)	114 (43)	143 (54)	
Girls	313 (54)	15 (5)	152 (49)	146 (47)	
Ethnicity, n (%), *n* = 571					< 0.0001
European	231 (41)	16 (7)	143 (62)	72 (31)	
Māori	59 (10)	0 (0)	20 (34)	39 (66)	
Pacifica	122 (21)	2 (2)	21 (24)	99 (75)	
Asian^c^	138 (24)	5 (4)	61 (44)	72 (52)	
Others^d^	21 (4)	0 (0)	9 (43)	12 (57)	
School deciles, n (%)					< 0.0001
Low	147 (26)	1 (1)	26 (18)	120 (81)	
Medium	192 (33)	7 (4)	86 (45)	99 (51)	
High	239 (41)	15 (6)	154 (64)	170 (30)	
School policy					< 0.0001^e^
Water-only	478 (83)	14 (3)	204 (43)	260 (54)	
Not water-only	100 (17)	9 (9)	62 (62)	29 (29)	
**Anthropometric measures,** ***n*** **= 561**					
Height (m)	1.4 (1.4, 1.5)	1.4 (1.3, 1.5)	1.4 (1.4, 1.5)	1.4 (1.4, 1.5)	0.09
Weight (kg)	36 (31, 44)	34 (29, 35)	35 (30, 40)	37 (32, 47)	< 0.01
BMI (kg/m^2^)	17 (16, 20)	16 (16, 19)	17 (16, 19)	18 (16, 21)	< 0.0001
WC (cm)	60 (56, 67)	58 (56, 65)	58 (54, 63)	63 (57, 70)^b^	< 0.0001
% Body fat^f^	21 (16, 28)	19 (14, 27)	19 (15, 25)	22 (17, 30)^b^	< 0.0001
Lowest, < 21%, n (%)	301 (54)	16 (5)	163 (54)	122 (41)	< 0.0001
Highest, ≥21%, n (%)	260 (46)	7 (3)	94 (36)	159 (61)	

Values are median (25th, 75th percentiles), unless otherwise stated

*BMI* body mass index, *SD* standard deviation, *WC* waist circumference

^a^Kruskal-Wallis tests for continuous variables and Pearson’s chi-squared for categorical variables

^b^Compared to the first category (less than one serving of sugar-sweetened beverages per week), significantly different at *P* < 0.05

^c^Includes: South East Asian (Indonesian, Thai, Singaporean, Malaysian, Pilipino, and Laotian), South Asian (Indian, Pakistani, Sri Lankan, and Bangladeshi) and East Asian (Chinese, Taiwanese, Korean, and Japanese)

^d^Includes: Middle Eastern, Latin American, and African

^e^There was no association when the analysis was adjusted for school decile, *P* > 0.05

^f^Categorised based on the median BF% of population

### Sugar-sweetened beverage consumption and selected socio-demographic associates/correlates

The frequency of consumption of different beverages is presented in Table 3. SSBs, sugar containing carbonated beverages, and sugar-sweetened milk-based beverages were reported to be consumed at least once a week by 96% (46% 1-4 servings/week and 50% ≥5 servings/week), 39 (33% 1-4 servings/week and 6% ≥5 servings/week), and 85% (61% 1-4 servings/week and 24% ≥5 servings/week) of children, respectively. The proportions of children who reported having < 1, 1 and ≥ 2 servings a day were 42, 36, 22% for SSBs, 92, 6, and 2% for sugar containing carbonated beverages, and 70, 23, and 7% for sugar containing milk-based beverages, respectively.

**Table 3:** Frequency consumption of different beverages in 578 children

Beverages^**a**^, n (%)	Beverage consumption		
	< 1 serving/week	1-4 servings/week	≥5 servings/week
All sugar-sweetened beverages^b^	23 (4)	266 (46)	289 (50)
Sugar containing carbonated beverages^c^	353 (61)	191 (33)	34 (6)
Sugar-sweetened milk-based beverages^d^	90 (16)	352 (61)	136 (24)
Flavoured milk	454 (79)	94 (16)	30 (5)
Milk drinks	450 (78)	103 (18)	25 (4)
Milk drink made from powder	315 (55)	190 (33)	73 (13)
Fruit drink made from powder	483 (84)	64 (11)	31 (5)
Fruit drink made from concentrate	451 (78)	97 (17)	30 (5)
Soft/fizzy drink	405 (70)	146 (25)	27 (5)
Soda stream drink	540 (93)	29 (5)	9 (2)
Energy drink	567 (98)	8 (1)	3 (1)
Sport drink	543 (94)	29 (5)	6 (1)
Flavoured water	531 (92)	33 (6)	14 (2)
Sugar-sweetened tea/coffee	428 (74)	103 (18)	47 (8)
Fruit juice	348 (60)	181 (31)	49 (9)
Fruit smoothie	463 (80)	94 (16)	21 (4)

^a^Eighty-four children consumed other beverages not listed in the beverage consumption questionnaire (including sparkling water, coconut water, Yakult, iced tea, homemade honey water, and kombucha), of whom 31, 56 and 13% consumed < 1, 1-4, and ≥ 5 servings of those beverages per week

^b^Fruit juice and smoothies were excluded from SSBs because children cannot distinguish fruit juice from 100% fruit juice and whether fruit smoothies contain added sugar or not

^c^Soft/fizzy drinks + soda stream drinks + energy drinks

^d^Flavoured milk + milk drinks (ready and made from powder)

Among SSBs, milk drinks made from powder, fruit juice and/or soft/fizzy drinks were reported to be consumed each at least once a week by 30-46% (25-33% 1-4 servings/week and 5-13% ≥5 servings) of children. A larger proportion of children of Māori, Pacific, Asian and other ethnicities (than Europeans; > 65% vs. 43%, *P* < 0.0001), and children of low and medium decile schools (than high decile schools; 90 and 63% vs. 43%, *P* < 0.0001) reported consuming at least five servings of SSBs a week (Table 2). The association of sex, ethnicity and school deciles with SSBs consumption was independent of the effect of one another.

### Home/community/school environment and their association with beverage consumption

More than 20% of children reported having milk drinks, fruit smoothies and fruit juice available at home usually/always. Other beverages were less often available at homes (never/sometimes) (Table 4). The availability of all beverages (except for fruit drinks) at home (usually/always) was associated with higher consumption of beverages (Table 4).

**Table 4:** Home availability of different beverages and its association with consumption^a^

Home availability of beverages, n (%)	Total	Beverage consumption			
		< 1 Serving/week	1-4 Servings/week	≥5 servings/week	***P***-value^**b**^
Flavoured milk					< 0.0001
Never/sometimes	503 (91)	425 (84)	65 (13)	13(3)	
Usually/always	53 (9)	15 (28)	25 (47)	13 (25)	
Milk drink					< 0.0001
Never/sometimes	348 (62)	291 (84)	47 (14)	10 (3)	
Usually/always	210 (38)	145 (69)	53 (25)	12 (6)	
Fruit drink					0.47
Never/sometimes	496 (88)	418 (84)	53 (11)	23 (5)	
Usually/always	65 (12)	51 (79)	9 (14)	5 (8)	
Fruit cordial					< 0.0001
Never/sometimes	492 (87)	403 (82)	76 (15)	13 (3)	
Usually/always	71 (13)	38 (54)	20 (28)	13 (18)	
Soft drink					< 0.0001
Never/sometimes	515 (91)	386 (75)	119 (23)	10 (2)	
Usually/always	47 (9)	11 (23)	20 (43)	16 (34)	
Fruit juice					< 0.0001
Never/sometimes	483 (87)	319 (66)	142 (29)	22 (5)	
Usually/always	73 (23)	15 (21)	35 (48)	23 (32)	
Fruit smoothie					< 0.0001
Never/sometimes	402 (72)	346 (86)	49 (12)	7 (2)	
Usually/always	155 (28)	102 (66)	39 (25)	14 (9)	

^a^Some children did not answer all questions regarding home availability of beverages; therefore, the numbers do not add up to 578

^b^Pearson’s chi-squared test (significant at *P* < 0.05)

Beverages were consumed at least once a week outside of the home by 45% of children. Beverages were purchased from fast food outlets, dairies/petrol stations and supermarkets at least once a week by 43, 13, and 45% of the children, respectively. Visiting fast food outlets, dairies and supermarkets was associated with higher consumption of SSBs and sugar containing carbonated beverages (≥1 serving a week, *P* < 0.05).

A large proportion of children (74%) reported their schools encouraged them to consume healthy beverages a lot (20 and 6% were encouraged sometimes and not at all, respectively). Schools’ healthy beverage policies (encouragement and water-only policy) were not associated with beverage consumption (*P* > 0.05, adjusted for school decile).

### Children’s reasoning and knowledge/attitude towards beverage consumption

A large proportion of children reported satisfying thirst (66%) and taste (59%) as the reasons for choosing a beverage. Also, children chose beverages because they were perceived to be healthier (27%), allowed at home/school (22%) and affordable (17%). Affordability significantly influenced consumption of ≥1 serving of sugar containing carbonated drinks, soft/fizzy drinks and milk drinks a week in 53, 43, and 31% of children, respectively. Furthermore, satisfying thirst significantly influenced the consumption of ≥1 serving of soft/fizzy drink a week in 33% of children. No association was found between other reasons and beverage consumption.

The most and least healthy beverages reported by children were water (92%) and soft/fizzy drinks (97%), respectively. Children’s knowledge about which beverages are SSBs and associations with consumption is presented in Table 5. A large proportion of children correctly identified flavoured milk (86%), milk drinks (81%), soft/fizzy drinks (95%), and energy drinks (87%) as being SSBs. The ability to accurately identify drinks as SSBs did not impact consumption, as approximately 60% of those who correctly identified SSBs also reported consuming ≥5 servings of SSBs a week.

**Table 5:** Children’s knowledge about which beverages are sugar-sweetened beverages and associations with consumption^a^

Beverages^**b**^, n (%)	Total	Beverage consumption		***P***-value
		< 5 servings/week	≥5 servings/week	
Flavoured milk				0.20
Correct	474 (86)	188 (40)	286 (60)	
Incorrect/do not know	78 (14)	25 (32)	53 (68)	
Milk drinks				0.07
Correct	450 (81)	81 (40)	269 (60)	
Incorrect/do not know	107 (19)	33 (31)	74 (70)	
Soft/fizzy drinks				0.06
Correct	528 (95)	207 (39)	321 (61)	
Incorrect/do not know	28 (5)	6 (21)	22 (79)	
Sports drinks				< 0.01
Correct	416 (75)	174 (42)	242 (58)	
Incorrect/do not know	137 (25)	38 (28)	99 (72)	
Energy drinks				0.06
Correct	484 (87)	192 (40)	292 (60)	
Incorrect/do not know	71 (13)	20 (28)	51 (72)	

^a^Some children did not answer all questions in the knowledge/attitude towards beverages questionnaire; therefore, the numbers do not add up to 578 (whose age and sex were available and who answered all questions in the sugar-sweetened beverage type and frequency questionnaire)

^b^Data regarding children’s knowledge about fruit juice and smoothie were collected, but because children cannot distinguish fruit juice from 100% fruit juice and whether fruit smoothie contain added sugar or not, they are not reported

Children’s perception of adequate beverage consumption is presented in Table 6. Except for milk drinks and tea/coffee, approximately 90% of children correctly recognised that SSBs should not be consumed everyday/most days. However, 15% of children believed that milk drinks should be consumed daily. Children who believed that SSBs could be consumed every/most days were more likely to consume ≥5 servings of drinks such as sports drinks and flavoured milk per week than those who selected sometimes or never.

**Table 6:** Children’s perception of adequate beverages consumption^a^

	Frequency			
Beverages ^**b**^, n (%)	Everyday/most days	Sometimes/occasionally	Never	Do not know
Flavoured milk	18 (3)	326 (74)	132 (23)	4 (1)
Milk drinks	85 (15)	391 (68)	91 (16)	9 (2)
Soft/fizzy drinks	10 (2)	459 (79)	106 (18)	3 (1)
Sports drink/sports water/flavoured water	16 (3)	303 (53)	238 (41)	17 (3)
Energy drink	1 (0.2)	63 (16)	472 (82)	12 (2)

^a^Some children did not answer all questions in the knowledge/attitude towards beverages questionnaire; therefore, the numbers do not add up to 578 (whose age and sex were available and who answered all questions in the sugar-sweetened beverage type and frequency questionnaire)

^b^Data regarding children’s perception of adequate fruit juice and smoothie consumption were collected, but because children cannot distinguish fruit juice from 100% fruit juice and whether fruit smoothie contain added sugar or not, they are not reported

### Sugar-sweetened beverages consumption and associations with BF%

A larger proportion of children having BF% ≥21% than < 21% consumed ≥5 servings of SSBs a week, 57% vs. 43%, OR = 1.6 95% CI 1.3, 2.7, *P* = 0.01 (adjusted for sex and school decile, Table 7). Further analysis revealed that girls, but not boys (*P* > 0.05), consuming ≥5 servings of SSBs a week had 2.4 times increased odds (adjusted for school decile) of being in the highest BF% category, compared to those consuming < 5 servings a week (*P* < 0.01). The odds (adjusted) of being in the highest BF% category increased by 1.7 and 2.6 times in girls who consumed ≥1 servings of sugar containing carbonated beverages and soft drinks, respectively compared with < 1 serving a week.

**Table 7:** The relationship between sugar-sweetened beverages and BF% in 561 children

Variables^**a**^	Lower BF% (< 21%) n (%) = 301 (54)	Higher BF% (≥21%) n (%) = 260 (46)	Association with BF%^**b**^
			Multivariable OR (95% CI)
Sex			
Boys	159 (62)	97 (38)	Reference category
Girls	142 (47)	163 (53)	2.1 (1.5. 3.0)
School deciles			
Low	52 (36)	94 (64)	Reference category
Medium	102 (53)	89 (47)	0.6 (0.4, 0.9)
High	147 (66)	77 (34)	0.4 (0.2, 0.6)
Sugar-sweetened beverages (servings per week)^c^			
< 5 servings	179 (64)	101 (36)	Reference category
≥ 5 servings	122 (43)	159 (57)	1.6 (1.3, 2.7)^d^

*CI* confidence Interval, *BF%* body fat percentage, *OR* Odds Ratio

^a^Model *x*^*b*^ (4) = 57, *P* < 0.0001

^b^BF% was coded as lower vs. higher: 1 = lower BF% (< 21%) and 2 = higher BF% (≥21%)

^c^At the univariate level, a larger proportion of children consuming at least 5 servings of sugar-sweetened beverages were within the highest BF% category, 2.3 (1.6, 3.2), *P* < 0.0001

^d^*P <* 0.01; The model was adjusted for sex and school deciles

## Discussion

In this study, most children reported consuming ≥1 serving of SSBs a week, of whom, approximately two thirds consumed ≥5 servings of SSBs a week. Consumption of SSBs was associated with several socio-demographic and environmental factors. Being of non-European ethnicity, or from low decile schools was associated with a greater consumption of SSBs. Home availability of SSBs and visiting fast foods/dairies/supermarkets were associated with a greater consumption of some beverages. Also, children’s perception of how frequently different beverages should be consumed was associated with their consumption. We also identified an association of SSBs with BF%, with the odds of having higher BF% increased in girls who consumed ≥5 servings of SSBs a week.

It is difficult to compare prevalence of beverage consumption across studies due to differences in study design and the way SSBs consumption levels are measured and reported (e.g. by volume, frequency per day or week, percentage of total beverage intake or of total energy intake). There is limited New Zealand children-level data on SSBs consumption patterns and levels; nevertheless, prevalence of weekly SSBs consumption (≥5 servings) reported in this study was well above the 2002 NZ National Children’s Nutrition Survey (50% vs. approximately 33% of children aged 5-14 years) [16]. However, our results regarding the consumption of particular beverages including sugar containing carbonated beverages and fruit juice are in line with previous surveys in New Zealand children and adolescents [17, 27]. A study pooling individual children and adolescent data of 13 cross-sectional surveys each from different countries showed that there is significant heterogeneity both across and within regions, although there are trends of high soft beverages and fruit juice consumption (even higher than water intake) in some countries [28].

In line with studies from NZ and other countries [29, 30], we found children of some ethnic groups (e.g. Pacific, Māori, and Asian, as compared to European) were more likely to consume SSBs more frequently. Ethnic groups in our study were disproportionally distributed across different school deciles, with a larger proportion of Māori (73%), Pacific (84%), and Asian (81%) than European children (26%) being from low and medium decile schools. Socially disadvantaged children and adolescents have been consistently shown to have greater SSBs consumption [30–32], and our findings provided further support for the existing evidence. Despite having water-only policy, children in the present study who attended lower decile schools were more likely to consume SSBs more frequently (at least five times a week) compared to children who attended high decile schools (data not shown).

Some school-based interventions have been shown to be effective in reducing SSBs consumption in children and adolescents [32, 33]. A systematic review of 36 studies investigating 36 different interventions (educational/behavioural, legislative/environmental or both) showed that 70% of all interventions were effective in decreasing SSBs consumption in adolescents, with legislative/environmental studies showing the highest success rate (90%) [33]. However, our findings contradicted previous evidence because a water-only policy and school encouragement were not associated with SSBs consumption (adjusted for school decile). Watts et al. [34] also showed that school explained only 1% of variance in SSBs consumption in adolescents. It is important to note that school policy is one of the multi-contextual factors (e.g. peer pressure, personal, home/family, and environmental factors) that may contribute to SSBs consumption in children, and other factors should also be taken into consideration. For example, previous studies have shown a relationship between greater proximity and diversity of food outlets to home and school and children’s and adolescents’ food purchasing and consumption, including SSBs [31, 35–37]. We also showed that more frequent fast foods/dairies/supermarket visits were associated with increased SSBs consumption, a finding confirmed by others [36]. There is a higher concentration of convenience and takeaway stores in low decile areas in New Zealand [38]. These findings highlight the importance of community environment as a contributing factor to SSBs consumption in children.

Evidence repeatedly shows that the availability and accessibility of foods and beverages in the home, are associated with food and beverage choice and consumption in children and adolescents [34, 39–42]. Consistent with previous studies [31, 43, 44], home availability of almost all SSBs assessed in this study (including fruit-based drinks and soft/fizzy drinks) was associated with their higher consumption. Accordingly, there is an important opportunity for health professionals to encourage parents to improve children’s healthy beverage consumption by modifying their purchasing habits (limiting the purchase of SSBs). This might be concerning for some countries like New Zealand where SSBs price is much lower than that of healthy unsweetened beverages (e.g. plain milk). In addition, we found an association between affordability and greater SSBs consumption, a finding confirmed by others [45]. These findings coupled with the known price incentive purchase [46] supports interventions that target the price of SSBs (through taxation [47] and income supplements such as increased minimum wage) to support families being able to afford other foods.

Our findings showed a large proportion of children had a good knowledge regarding SSBs, a finding consistent with a previous study among children [23]. However, there were some beverage knowledge gaps in children; e.g. some children perceived that milk drinks (which are sweetened) should be consumed daily.

Although perception of adequate beverage consumption frequency was related to the consumption, no association was found between correct identification of SSBs and consumption. It is important to note that perception and knowledge do not necessarily predict beverages consumption behaviour; for example, while Park et al. [48] found no association between health-related knowledge and SSBs consumption, Kim et al. [49] found a better health-related knowledge was associated with worse beverage consumption (higher consumption of SSBs). Furthermore, the findings of these studies (including the present study) may not be comparable and generalisable as they are conducted in different age groups (children and adults) and countries (US and New Zealand), and different measures were used to assess study population’s knowledge (e.g. identifying SSBs and their consumption frequency, understanding the composition of drinks such as energy and sugar, and potential health effects associated with consumption). The knowledge/attitude questions used in the present study were not validated in children, and there might have been some limitations with some questions (e.g. questions about fruit juice and smoothie). Previous evidence suggest that educational/behavioural interventions are effective in reducing SSBs consumption in adolescents and adults [33, 50]. Accordingly, efforts to reduce SSBs consumption in children might benefit from the inclusion of educational interventions that empower children and parents/caregivers (due to many reasons; e.g. having control over the home availability of beverages and role modelling) to make healthy choices [32, 42, 51, 52]. To assess SSBs knowledge/attitude of children and adults and the efficacy of educational interventions, it is of particular importance to develop and validate age-specific SSBs knowledge/attitude questionnaires.

There is strong evidence to support a positive relationship between SSBs consumption and weight status, BMI and/or body fat in children, and interventions that can lead to changes in body fatness in children by reducing the consumption of SSBs [10, 53]. In line with these studies, our findings showed that BF% was positively associated with SSBs consumption in children. However, the association was observed only in girls. Previous studies have provided mixed results regarding the effect of sex on SSBs and measures of adiposity association in children. While one study showed a higher soft drink consumption was associated with BMI z score in boys [54], another study failed to find an effect of sex interaction on the relationship [55]. It is important to note that there are some sex differences in the lifestyle risk factors of high BF% that were not measured and controlled for in the present study (e.g. other dietary factors and physical activity) [29]. Also, sexual maturation/pubertal status could influence children’s BF%. Although both sexes experience rapid increases in body fat, the proportion of increase is slower in boys than girls [56]. Future studies should comprehensively measure and control for obesogenic behaviours and pubertal status when investigating the association between SSBs consumption and BF%.

This study has several strengths and limitations that warrant discussion. The major strength of this study is that it examined data from a relatively large sample of New Zealand school age children, including a relatively large number of children across a range of ethnicities which, with the exception of Māori, were similar in proportion to the NZ population. However, the method of recruitment (a convenience sample living in Auckland) limits the generalisability of this study. Furthermore, cross-sectional data means that it is difficult to infer causality from the associations observed. Also, the data was collected during one season (winter), and seasonal differences in SSBs consumption were not assessed. The consumption of SSBs might have been underestimated, given that a large proportion of children indicated consuming SSBs to satisfy thirst and that the climate of the two seasons, especially summer and winter, are different in New Zealand. It is important to note that assessing beverages consumption in children is associated with unique challenges, mainly related to the ongoing cognitive development of children, limited literacy skills, and difficulty in estimating portion sizes. Although we aimed to address these challenges in the current study by using survey methodologies which were required to be completed by children with and without the help of their parents, using simple definitions of drinks that are appropriate for children (e.g., fruit juice rather than 100% fruit juice and fruit juice with added sugar or fruit smoothie rather than fruit smoothie with added sugar), including pictures of beverages, and using brief measures of recall limited to pre-determined drinks (which are not as comprehensive as food diary methodology), errors in estimating beverages consumption in children cannot be ruled out. Also, the methods of estimating weekly/daily servings could have resulted in underestimation of beverage consumption. Finally, due to inadequate statistical power (having only two schools without water-only policy), caution should be practiced when interpreting the results regarding the association of school policy with SSBs consumption.

## Conclusions

In conclusion, many New Zealand children in this cohort regularly consume SSBs, with higher consumption rates observed in those from socially disadvantaged areas, and those reported having them available at home usually/always. This high consumption is associated with higher adiposity in children. Thus, multi-contextual interventions to decrease SSBs intake should target this population and their families/environment. Because beverage consumption is strongly influenced by home availability, educational programs must include the whole family, especially the person who does the shopping.

## Supplementary Information

**Additional file 1.**

## Data Availability

The datasets used and/or analysed during the current study are available from the corresponding author on reasonable request.

## Abbreviations

BF%: Body fat percentage
BIA: Bioelectrical impendence
CI: Confidence interval
MOH: Ministry of Health
NZ: New Zealand
OR: Odd ratio
SSBs: Sugar-sweetened beverages
WHO: World Health Organisation
