# Supplementary material for: Sugar-sweetened beverages consumption among New Zealand children aged 8-12 years: a cross sectional study of sources and associates/correlates of consumption
Source: BMC Public Health. 2021 Dec 13;21:2277. doi: 10.1186/s12889-021-12345-9 (PMC8670206; doi:10.1186/s12889-021-12345-9)
Supplement: Supplementary file 1 — Additional file 1. [file 12889_2021_12345_MOESM1_ESM.docx]

**Beverages Consumption among New Zealand Children Aged 8-12 Years: A Cross Sectional Study of Sources and Associates/Correlates of Consumption**

Emma Smirk^1^, Hajar Mazahery^1^, Cathryn A. Conlon^1^, Kathryn L. Beck^1^, Cheryl Gammon^2^, Owen Mugridge^1^, Pamela R. von Hurst^1*^

^1^School of Sport, Exercise and Nutrition, College of Health, Massey University, Auckland 0745, New Zealand

^2^School of Health Sciences, College of Health, Massey University, Auckland 0745, New Zealand

*Corresponding author: Prof. Pamela R. von Hurst; Phone: +64 (09) 2136657; Email: p.r.vonhurst@massey.ac.nz

**Supplementary Material 1**

**Beverage Type and Frequency Questionnaire**

**Complete this questionnaire with help from your Mum, Dad or other family member.**

**Put a tick √ in the box which best tells HOW OFTEN you eat the drinks listed below (one glass serving size)**

1. **Milk (not flavoured)**

| Never or less than once a month | 1-3 times a month | 1-2 times a week | 3-4 times a week | 5-6 times a week | Once a day | 2 or more times a day |
| --- | --- | --- | --- | --- | --- | --- |
|  |  |  |  |  |  |  |

1. **What kind of milk do you usually drink? Place a tick √ in the box**

| Standard milk (dark blue) | Trim (green) | Mega Milk (orange) |
| --- | --- | --- |
| Low Fat (light blue) | Extra calcium (yellow) | Other milk (please name below) |

1. **Flavoured milk**

| Never or less than once a month | 1-3 times a month | 1-2 times a week | 3-4 times a week | 5-6 times a week | Once a day | 2 or more times a day |
| --- | --- | --- | --- | --- | --- | --- |
|  |  |  |  |  |  |  |

1. **Milk shake or milk drink eg Up and Go**

| Never or less than once a month | 1-3 times a month | 1-2 times a week | 3-4 times a week | 5-6 times a week | Once a day | 2 or more times a day |
| --- | --- | --- | --- | --- | --- | --- |
|  |  |  |  |  |  |  |

1. **Fruit Smoothie eg Simply Squeezed, Meadow Fresh Yoghurt Smoothie**

| Never or less than once a month | 1-3 times a month | 1-2 times a week | 3-4 times a week | 5-6 times a week | Once a day |  | 2 or more times a day |
| --- | --- | --- | --- | --- | --- | --- | --- |
|  |  |  |  |  |  |  |  |

1. **Flavoured milk drink made from powder eg Milo, Nesquik, hot chocolate**

| Never or less than once a month | 1-3 times a month | 1-2 times a week | 3-4 times a week | 5-6 times a week | Once a day | 2 or more times a day |
| --- | --- | --- | --- | --- | --- | --- |
|  |  |  |  |  |  |  |

**6a. With flavoured milk drinks made from powder eg Milo, Nesquik or hot chocolate, do you use? Place a tick √ in the box**

| All milk | ½ milk | 1/4 or less milk |
| --- | --- | --- |
|  |  |  |

**6b. With flavoured milk drinks made from powder eg Milo, Nesquik or hot chocolate, do you add sugar? Place a tick √ in the box**

| Yes | No | Not applicable |
| --- | --- | --- |
|  |  |  |

1. **Juice eg fresh orange juice, juices such as McCoys, Robinsons, Keri**

| Never or less than once a month | 1-3 times a month | 1-2 times a week | 3-4 times a week | 5-6 times a week | Once a day | 2 or more times a day |
| --- | --- | --- | --- | --- | --- | --- |
|  |  |  |  |  |  |  |

1. **Powdered fruit drink eg Refresh, Raro**

| Never or less than once a month | 1-3 times a month | 1-2 times a week | 3-4 times a week | 5-6 times a week | Once a day | 2 or more times a day |
| --- | --- | --- | --- | --- | --- | --- |
|  |  |  |  |  |  |  |

1. **Fruit drink from concentrate or cordial eg Just Juice, Ribena**

| Never or less than once a month | 1-3 times a month | 1-2 times a week | 3-4 times a week | 5-6 times a week | Once a day | 2 or more times a day |
| --- | --- | --- | --- | --- | --- | --- |
|  |  |  |  |  |  |  |

1. **Standard soft drinks or other fizzy drinks eg Coke, Lemonade, Fanta, Mountain Dew**

| Never or less than once a month | 1-3 times a month | 1-2 times a week | 3-4 times a week | 5-6 times a week | Once a day | 2 or more times a day |
| --- | --- | --- | --- | --- | --- | --- |
|  |  |  |  |  |  |  |

1. **Soda stream**

| Never or less than once a month | 1-3 times a month | 1-2 times a week | 3-4 times a week | 5-6 times a week | Once a day | 2 or more times a day |
| --- | --- | --- | --- | --- | --- | --- |
|  |  |  |  |  |  |  |

1. **Diet drinks / Artificially sweetened drinks eg Diet Coke, Coke Zero**

| Never or less than once a month | 1-3 times a month | 1-2 times a week | 3-4 times a week | 5-6 times a week | Once a day | 2 or more times a day |
| --- | --- | --- | --- | --- | --- | --- |
|  |  |  |  |  |  |  |

1. **Energy drinks eg V, Red Bull, Monster, Demon**

| Never or less than once a month | 1-3 times a month | 1-2 times a week | 3-4 times a week | 5-6 times a week | Once a day | 2 or more times a day |
| --- | --- | --- | --- | --- | --- | --- |
|  |  |  |  |  |  |  |

1. **Sports drinks eg Gatorade, Powerade, E2**

| Never or less than once a month | 1-3 times a month | 1-2 times a week | 3-4 times a week | 5-6 times a week | Once a day | 2 or more times a day |
| --- | --- | --- | --- | --- | --- | --- |
|  |  |  |  |  |  |  |

1. **Plain Water including tap water or bottled water like Pump, H2go, Water for Everyone water**

| Never or less than once a month | 1-3 times a month | 1-2 times a week | 3-4 times a week | 5-6 times a week | Once a day | 2 or more times a day |
| --- | --- | --- | --- | --- | --- | --- |
|  |  |  |  |  |  |  |

1. **Flavoured water / Vitamin Water like Mizone, H2go Zero water, Zero (flavoured) Water, Coconut water, Mizone, Loaded water**

| Never or less than once a month | 1-3 times a month | 1-2 times a week | 3-4 times a week | 5-6 times a week | Once a day | 2 or more times a day |
| --- | --- | --- | --- | --- | --- | --- |
|  |  |  |  |  |  |  |

1. **Tea**

| Never or less than once a month | 1-3 times a month | 1-2 times a week | 3-4 times a week | 5-6 times a week | Once a day | 2 or more times a day |
| --- | --- | --- | --- | --- | --- | --- |
|  |  |  |  |  |  |  |

**17a. If you drink tea, do you add milk? Place a tick √ in the box**

| Yes | No | Not applicable |
| --- | --- | --- |
|  |  |  |

**17b. If you drink tea, do you add sugar? Place a tick √ in the box**

| Yes | No | Not applicable |
| --- | --- | --- |
|  |  |  |

1. **Coffee**

| Never or less than once a month | 1-3 times a month | 1-2 times a week | 3-4 times a week | 5-6 times a week | Once a day | 2 or more times a day |
| --- | --- | --- | --- | --- | --- | --- |
|  |  |  |  |  |  |  |

**18a. If you drink coffee, do you add milk? Place a tick √ in the box**

| Yes | No | Not applicable |
| --- | --- | --- |
|  |  |  |

**18b. If you drink coffee, do you add sugar? Place a tick √ in the box**

| Yes | No | Not applicable |
| --- | --- | --- |
|  |  |  |

1. **“Other drinks” group. If you often have another drink that is not listed, give the name and tick √ how often you have it.**

**Drink __________________________________________________**

| Never or less than once a month | 1-3 times a month | 1-2 times a week | 3-4 times a week | 5-6 times a week | Once a day | 2 or more times a day |
| --- | --- | --- | --- | --- | --- | --- |
|  |  |  |  |  |  |  |

1. **During the last 7 days, how often did you drink any of the following?**

|  | None in last 7 days | 1-3 times a week | 4-5 times a week | Once a day | 2 or more times a day |
| --- | --- | --- | --- | --- | --- |
| 20a. Milk, plain/unflavoured |  |  |  |  |  |
| 20b. Chocolate milk or other flavoured milk |  |  |  |  |  |
| 20c. Standard soft drinks or other fizzy drinks (eg Coke, Sprite, Fanta) |  |  |  |  |  |
| 20d. Diet drinks / Artificially sweetened drinks eg Diet Coke, Coke Zero |  |  |  |  |  |
| 20e. Juice eg fresh orange juice, juices such as McCoys, Robinsons, Keri |  |  |  |  |  |
| 20f. Fruit drinks (Ribena, Raro, Just juice, etc - not 100% juice) |  |  |  |  |  |
|  | None in last 7 days | 1-3 times a week | 4-5 times a week | Once a day | 2 or more times a day |
| 20g. Fruit drink from concentrate or cordial eg Just Juice, Ribena |  |  |  |  |  |
| 20h. Tap water |  |  |  |  |  |
| 20i. Plain bottled water eg Pump, H2go |  |  |  |  |  |
| 20j. Flavoured water |  |  |  |  |  |

1. **During the last 7 days, did you get a drink from any of these places?**

|  | None in last 7 days | 1-3 times a week | 4-5 times a week | Once a day | 2 or more times a day |
| --- | --- | --- | --- | --- | --- |
| 21a. A fast food place (eg McDonalds, KFC, Burger King, Subway, Pizza Hut) |  |  |  |  |  |
| 21b. Other takeaways or fast-food shops (fish & chips, Chinese takeaways) |  |  |  |  |  |
| 21c. Dairy or petrol stations |  |  |  |  |  |
| 21d. Supermarket |  |  |  |  |  |
| 21e. Somewhere else |  |  |  |  |  |

1. **If you answered yes above, when did you get the drink? Place a tick √ in the box**

| Before school | After school | In the evening |
| --- | --- | --- |
| In the weekend | Other (please list) |  |

1. **Were you? Place a tick √ in the box**

| Alone | With friends | With parents or family |
| --- | --- | --- |
| With brother or sister | With other caregiver | Other (please list below) |

1. **How often are the following available to eat or drink at home?**

|  | Never | Sometimes | Usually | Always |
| --- | --- | --- | --- | --- |
| 24a. Milk |  |  |  |  |
| 24b Flavoured milk |  |  |  |  |
| 24c. Food milk drinks eg Milo, Nesquik, Up and Smoothies (purchased) |  |  |  |  |
| 24e. Smoothie (made at home) |  |  |  |  |
| 24f. Fizzy drinks or soft drinks eg Coke, Lemonade |  |  |  |  |
| 24g. Artificially sweetened or diet drinks eg Diet Coke, Coke Zero |  |  |  |  |
| 24h. Soda stream drink made at home |  |  |  |  |
| 24i. Fruit juice eg McCoys, Robinsons, Keri |  |  |  |  |
| 24j. Fruit drink eg Just Juice, Ribena |  |  |  |  |
| 24k. Fruit cordial eg Raro, Refresh |  |  |  |  |

**25. How much do you care about drinking healthy drinks? Place a tick √ in the box**

| Not a lot | Sometimes | A lot |
| --- | --- | --- |
|  |  |  |

1. **How much do your friends care about drinking healthy drinks? Place a tick √ in the box**

| Not a lot | Sometimes | A lot |
| --- | --- | --- |
|  |  |  |

**27. Are you encouraged to drink healthy drinks at home? Place a tick √ in the box**

| Not a lot | Sometimes | A lot |
| --- | --- | --- |
|  |  |  |

**28. How much does your school encourage you to drink healthy drinks? Place a tick √ in the box**

| Not a lot | Sometimes | A lot |
| --- | --- | --- |
|  |  |  |

**29. Tick √ whether you agree, disagree or don’t know with the following statements**

**29a. Drinking a sugar-sweetened drink increases my energy intake**

| Disagree | Agree | Don’t know |
| --- | --- | --- |
|  |  |  |

**29b. I feel full after drinking a sugar-sweetened drink**

| Disagree | Agree | Don’t know |
| --- | --- | --- |
|  |  |  |

**29c. I’ve seen an ad for sugar-sweetened drinks on tv or on a sign recently**

| Disagree | Agree | Don’t know |
| --- | --- | --- |
|  |  |  |

**Thank you very much for filling out this questionnaire.**

**Please check that you have answered all questions.**

**Have a great day! ☺**
